# Abortive Treatment of Gonorrhea

**Published:** 1915-11

**Authors:** 


**Abortive Treatment of Gonorrhea.** Robinson, American Medicine, April, 1915. The patient urinates and the anterior urethra is gently washed out with about four ounces of warm normal salt solution (7:1000). No force must be used, and not more than a drachm or two of solution should be at any time in the urethra (so as to prevent any fluid from opening the cut-off muscle and penetrating into the posterior urethra), and the meatus should not be tightly closed by the tip of the syringe, so that the fluid may flow freely back. A few drops (5 to 10) of a 4-per-cent solution of cocaine, eucaine, or alypin are then instilled into the urethra. One drachm of a 2 per-cent protargol solution is then injected; closing the meatus with the fingers, held in for five minutes. In three hours a drachm of a one-per-cent solution of protargol is injected and held in for three minutes. This injection—1 drachm of a one-per-cent protargol solution held in for three minutes—is repeated every two hours until four injections have been given. The next four injections, at three hour intervals, are given with one-half-per-cent solutions; and the next four injections, also at three-hour intervals, are given with one-fourth-per-cent solutions. If we use argyrol, the method is the same, only the strength of the solution is different. The initial solution is 50 per cent and the subsequent solutions 25 or 20 per cent.

The protargol and argyrol solutions may also be used alternately. The discharge is examined for gonococci every day. At the end of two or three days we know what we may expect. If the abortive treatment proves successful and the gonococci have disappeared or are becoming less and less, well and good. If not, then also well, though not so well; at any rate we have not hurt our patient, and we may then proceed with the regular treatment of acute gonococcal urethritis.

